# Neutrophil adhesion molecules in experimental rhinovirus infection in COPD

**DOI:** 10.1186/1465-9921-14-72

**Published:** 2013-07-08

**Authors:** Patrick Mallia, Simon D Message, Marco Contoli, Katrina K Gray, Aurica Telcian, Vasile Laza-Stanca, Alberto Papi, Luminita A Stanciu, Sarah Elkin, Onn M Kon, Malcolm Johnson, Sebastian L Johnston

**Affiliations:** 1 Airway Disease Infection Section, National Heart and Lung Institute, Imperial College London, Norfolk Place, London W2 1PG, UK; 2 Imperial College Healthcare NHS Trust, London, UK; 3 Research Centre on Asthma and COPD, University of Ferrara, Ferrara, Italy; 4 GlaxoSmithKline, Uxbridge, Middlesex, UK

**Keywords:** Chronic obstructive pulmonary disease, Exacerbations, Respiratory viruses, Neutrophils

## Abstract

**Background:**

COPD exacerbations are associated with neutrophilic airway inflammation. Adhesion molecules on the surface of neutrophils may play a key role in their movement from blood to the airways. We analysed adhesion molecule expression on blood and sputum neutrophils from COPD subjects and non-obstructed smokers during experimental rhinovirus infections.

**Methods:**

Blood and sputum were collected from 9 COPD subjects and 10 smoking and age-matched control subjects at baseline, and neutrophil expression of the adhesion molecules and activation markers measured using flow cytometry. The markers examined were CD62L and CD162 (mediating initial steps of neutrophil rolling and capture), CD11a and CD11b (required for firm neutrophil adhesion), CD31 and CD54 (involved in neutrophil transmigration through the endothelial monolayer) and CD63 and CD66b (neutrophil activation markers). Subjects were then experimentally infected with rhinovirus-16 and repeat samples collected for neutrophil analysis at post-infection time points.

**Results:**

At baseline there were no differences in adhesion molecule expression between the COPD and non-COPD subjects. Expression of CD11a, CD31, CD62L and CD162 was reduced on sputum neutrophils compared to blood neutrophils. Following rhinovirus infection expression of CD11a expression on blood neutrophils was significantly reduced in both subject groups. CD11b, CD62L and CD162 expression was significantly reduced only in the COPD subjects. Blood neutrophil CD11b expression correlated inversely with inflammatory markers and symptom scores in COPD subjects.

**Conclusion:**

Following rhinovirus infection neutrophils with higher surface expression of adhesion molecules are likely preferentially recruited to the lungs. CD11b may be a key molecule involved in neutrophil trafficking in COPD exacerbations.

## Introduction

Chronic obstructive pulmonary disease (COPD) is a growing global epidemic and its prevalence is expected to increase markedly in the future [1]. The major cause of morbidity and mortality in COPD are acute exacerbations that are associated with impaired quality of life, accelerated loss of lung function and enormous health care costs [2]. The main causes of exacerbations are viral and bacterial infections [3,4]. Acute exacerbations are associated with increased airways inflammation with an influx of inflammatory cells such as neutrophils into the airways [3,4] and this inflammatory response is believed to be central to the pathogenesis of exacerbations. Neutrophil migration from the blood is a tightly controlled process that involves interactions between adhesion molecules on neutrophils and their ligands on endothelial cells. Initial selectin-mediated rolling is followed by integrin-dependent firm adhesion and transendothelial migration. The selectins CD62L (L-selectin) and CD162 (P-selectin glycoprotein ligand-1) mediate the initial steps of rolling and capture, firm adhesion requires the integrins CD11a (lymphocyte function-associated antigen-1) and CD11b (macrophage associated antigen-1) and transmigration through the endothelial monolayer involves CD31 (platelet endothelial cell adhesion molecule-1) [5]. Other molecules including CD54 (intercellular adhesion molecule-1 (ICAM-1)) are also involved in neutrophil transmigration but their exact role is undetermined. In addition neutrophils express a number of activation molecules including CD63 and CD66b that are expressed on the cell surface upon activation and degranulation of neutrophils. The role of specific adhesion molecules in neutrophil recruitment *in vivo* in COPD has not been established. Neutrophilic inflammation in COPD is poorly responsive to corticosteroids, so different anti-inflammatory approaches are needed and inhibition of adhesion molecules is one such approach that merits exploration [6].

We have previously reported increases in sputum neutrophils in COPD subjects following experimental rhinovirus infection [4]. In the present study we analysed expression of adhesion molecules and activation markers on neutrophils in blood and sputum during experimental rhinovirus infection in COPD subjects and a control group of smokers with normal lung function.

## Methods

### Study participants

Subjects were recruited from Imperial College Healthcare NHS Trust (St Mary’s Hospital), the Royal Brompton Hospital, local General Practices and by advertisement. Ethical approval was obtained from St Mary’s Local Research Ethics Committee (study number 00/BA/459E). Two subject groups were recruited for this study – a group of COPD subjects (GOLD stage II) and a group of smokers with normal lung function. All subjects were aged between 40–75 years, non-atopic, were current or ex-smokers with at least 20 pack years cumulative smoking, had no symptoms of respiratory tract infection within the previous 8 weeks and no treatment with oral or inhaled corticosteroids, long-acting β-agonists or tiotropium. The clinical, inflammatory and virologic data from the experimental rhinovirus infection study have been reported in a previous publication [4].

At an initial screening visit potential subjects were assessed for their suitability for the study. For those entering the study baseline clinical sampling was performed 1–4 weeks prior to virus inoculation, which was on study day 0. 10 TCID_50_ of the virus was diluted in a total volume of 1 mL of 0.9% saline and inoculated in both nostrils. Blood and sputum were collected at baseline prior to infection, and on days 5, 9, 12, 15, 21, 28, 35 and 42 post-inoculation. Sufficient samples for complete analyses were collected from 10 smokers and 9 COPD subjects.

### Symptom scores

Symptoms were assessed using diary cards that were completed on a daily basis from screening until 6 weeks post-inoculation for the lower respiratory symptoms of shortness of breath, cough, wheeze, sputum quantity and sputum. The mean scores on days −6 to 0 were calculated and subtracted from subsequent daily scores to correct for baseline symptoms. In addition to the daily scores symptom scores were calculated in 2-week blocks. The *baseline phase* was the 2 weeks prior to infection and the 2-week period from day 1 to day 14 was termed the *acute phase*.

### Inflammatory mediators

Total numbers of inflammatory cells and neutrophils were counted in sputum samples and have been reported in our previous publication [4]. A number of inflammatory mediators including IL-6, IL-8, pentraxin and neutrophil elastase were measured in sputum supernatants using commercially available ELISAs and have been previously reported [4,7]. Peripheral blood cell counts were measured in the Clinical Haematology Laboratory of St Mary’s Hospital, Imperial College Healthcare NHS Trust.

### Isolation of neutrophils

Peripheral blood neutrophils were obtained according to published methods. Briefly whole blood was collected, layered onto a separation gradient (polymorphprep® Axis-Shield), centrifuged, the neutrophil layer aspirated and erythrocytes lysed. A sample of cells was stained with 0.1% trypan blue for counting and assessment of viability, the concentration adjusted to 4 × 10^6^/mL, 100 μL of this suspension added to the relevant antibodies, incubated at 4°C for 45 minutes, fixed with 1% paraformaldehyde and resuspended for analysis. Neutrophils prepared in this way were >90% pure and >90% viable.

Sputum was induced according to European Respiratory Society guidelines [8] and as previously described [4]. Subjects were pre-medicated with 200 μg salbutamol via metered dose inhaler and large volume spacer and baseline FEV_1_ measured. 4% saline was administered with a DeVilbiss UltraNeb99 ultrasonic nebuliser until an adequate sputum sample was obtained. Sputum was processed within 2 hours of induction. Sputum plugs were selected from saliva by macroscopic inspection of the sample and 0.1% Dithiothreitol (DTT) added in the ratio 4 ml DTT to 1 g sputum and the mixture agitated and filtered. The same volume PBS was added, the filtrate centrifuged and the supernatant aliquotted and stored at −80°C. The cell pellet was washed and resuspended and the cells counted to obtain total cell counts. Cells were then resuspended at 2 × 10^6^/mL and antibody staining performed as described for blood neutrophils.

### Flow cytometry

All cells were stained with conjugated antibodies (BD Pharmingen) and analyzed on a Fluorescence Activated Cell Sorter (Becton Dickinson). Gain and amplitude settings were consistent throughout the study for each subject. 20,000 events were counted for each sample of blood neutrophils, and between 5000 and 20,000 events for sputum neutrophils. Sputum cellular composition is heterogeneous consisting predominantly of neutrophils and monocyte/macrophages with smaller populations of epithelial cells, eosinophils and lymphocytes, therefore sputum required initial staining with CD14 (FITC) and CD45 (QR) staining to identify neutrophils. A gate was initially selected on size and granularity characteristics and cells were then further characterised with CD14 and CD45. Leukocytes were differentiated from cellular debris and epithelial cells by selecting CD45 (pan-leukocyte marker) positive cells and neutrophils differentiated from monocytes/macrophages by selecting CD14^dim^ cells [9,10]. This is described further in the Results section. The adhesion molecules CD11a, CD11b, CD62L, CD162, CD31, CD54 and the activation markers CD66b and CD63 were detected using phycoerythrin (PE)-labelled antibodies. PE-conjugated nonspecific antibodies of the same isotype as the receptor antibodies were used as controls to establish background fluorescence and nonspecific antibody binding. The (arithmetic) mean fluorescence intensity (MFI) of the cells stained with control antibody was subtracted from the MFI of the receptor antibodies to provide a measure of receptor-specific MFI. Cell surface marker expression on neutrophils was analysed using WinMDI 2. software.

### Statistical analysis

Data are presented as median (± interquartile range) and changes from baseline were analysed with the Friedman test. Differences between groups were analyzed by using Mann–Whitney tests. Correlations between data sets were examined using Spearman’s correlation. Differences were considered significant for all statistical tests at P values of less than 0.05 and all reported P values were two-sided. Analysis was performed using GraphPad Prism version 4.00 for Windows (GraphPad Software, San Diego, USA).

## Results

### Identification of neutrophils in sputum

**Figure 1:**
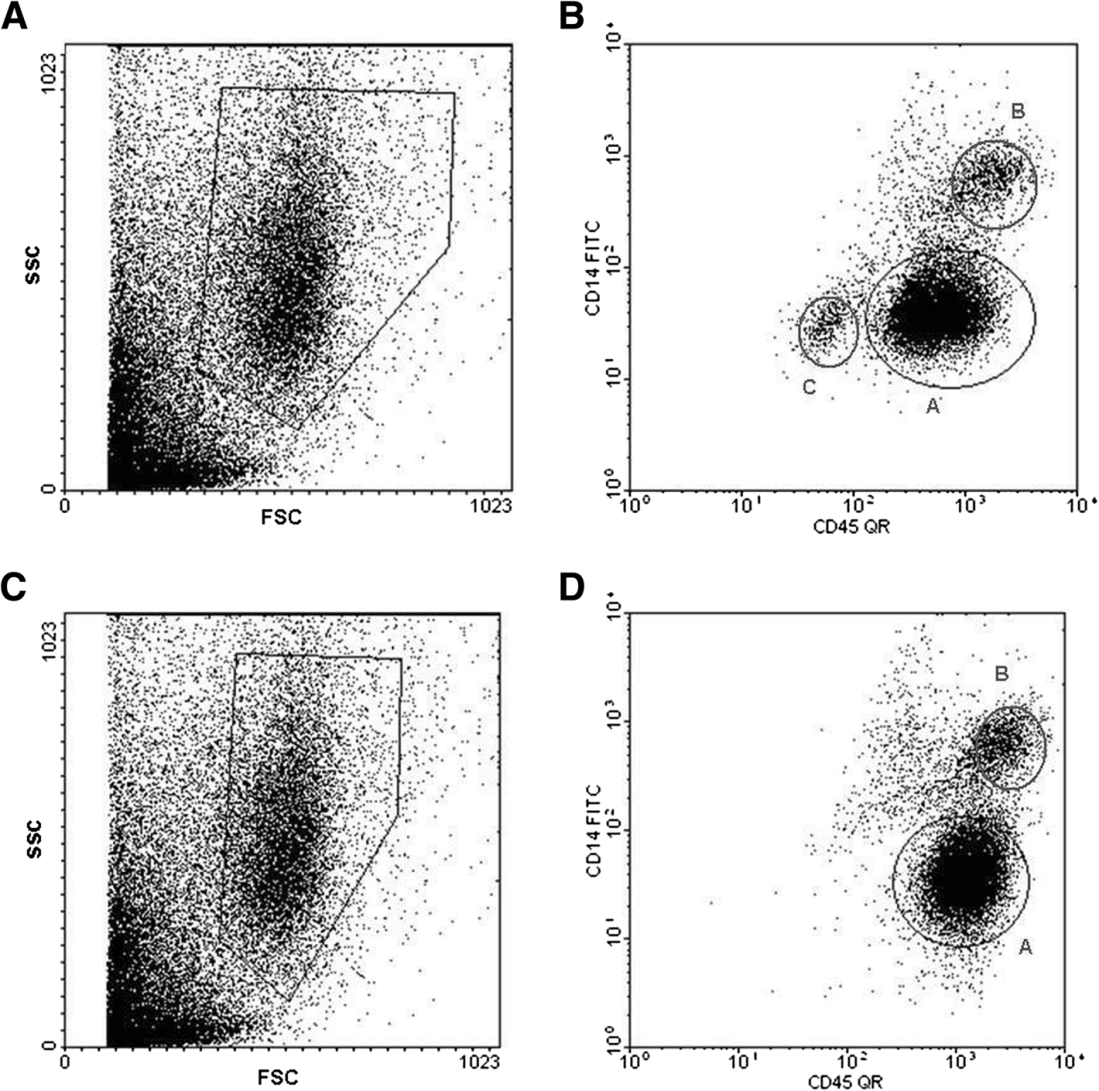
**Flow cytometry of sputum cells from 2 representative subjects. Panels A** and **C** show representative plots of unstained sputum. A gate was selected as shown to select for neutrophils on the basis of middle-range size and granularity. **Panels B** and **D** show these selected cells stained with the markers CD45 (pan-leukocyte marker) and CD14 (monocyte/macrophage marker). The CD14^low^, CD45^high^ cells (gate A) were selected as representing neutrophils and analysed for expression of neutrophil markers. Monocytes/macrophages (CD14^high^, CD45^high^ (gate B)) and epithelial cells (CD45^low^, CD14^low^ cells (gate C)) were excluded.

Neutrophils in sputum were identified using CD14 and CD45 staining as described in the Methods section. Representative plots of unstained cells showing size and granularity characteristics are shown in Figure 1A and 1C. Neutrophils were differentiated from cellular debris, epithelial cells and monocytes/macrophages by selecting CD45 positive/CD14^dim^ cells. Representative plots of CD45/CD14 stained cells are shown in Figure 1B and 1D.

### Neutrophil markers at baseline

Surface markers were initially measured on blood and sputum neutrophils at baseline prior to rhinovirus infection in the stable state. No significant differences in expression of any marker were found between the groups on either blood or sputum neutrophils (data not shown).

**Figure 2:**
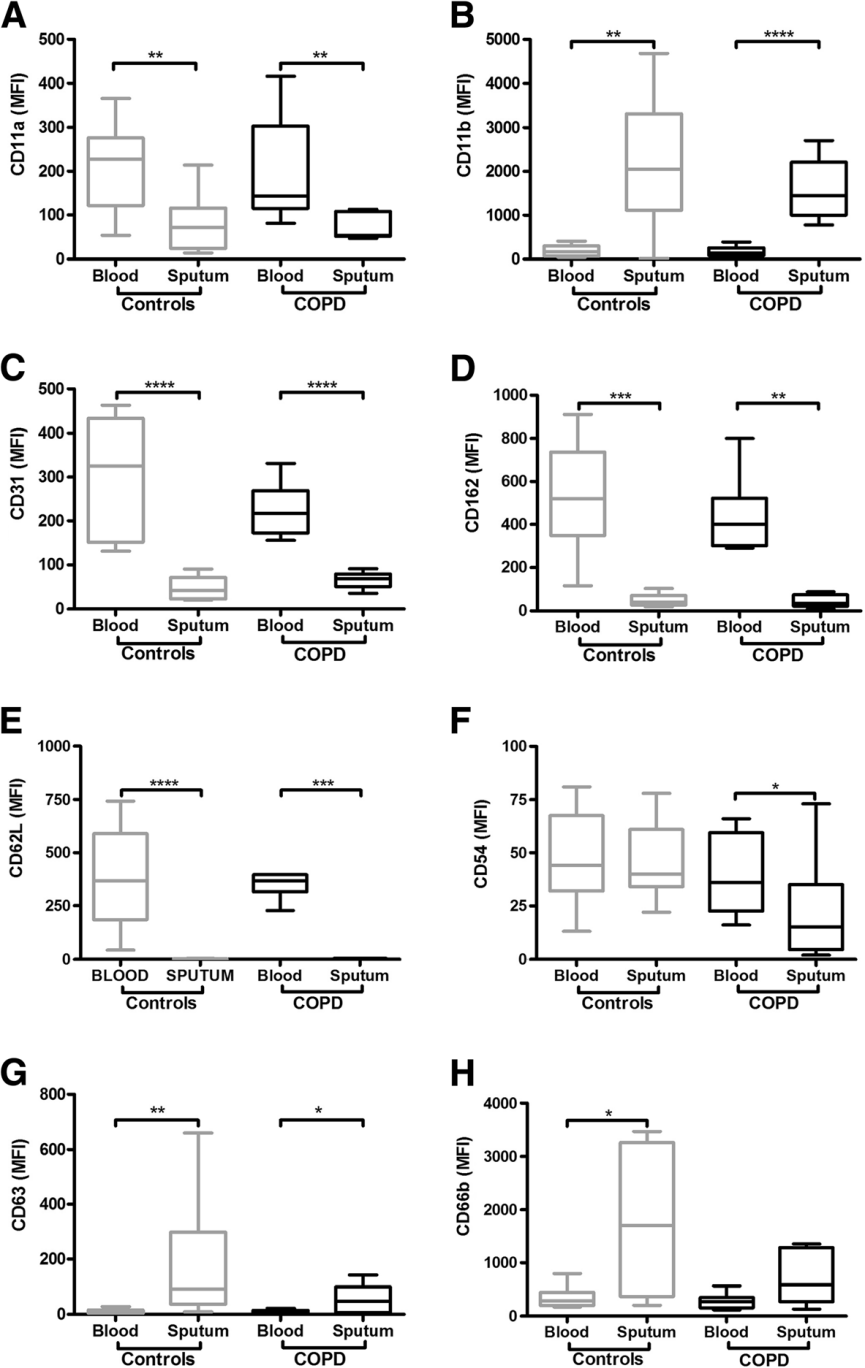
**Expression of neutrophil markers on blood and sputum neutrophils at baseline prior to infection in the COPD and control groups.** CD11a **(Panel A)**, CD11b **(Panel B)**, CD31 **(Panel C)**, CD162 **(Panel D)**, CD62L **(Panel E)**, CD54 **(Panel F)**, CD63 **(Panel G)**, CD66b **(Panel H)**.* P < 0.05, **P < 0.01, ***P < 0.001.

We next compared markers between blood and sputum neutrophils to analyse the effect of neutrophil migration on marker expression. Expression of CD11a, CD62L, CD31 and CD162 was significantly lower on sputum neutrophils compared to blood neutrophils in both groups (Figure 2), whereas CD11b and CD63 levels were significantly higher on sputum neutrophils. Expression of the activation marker CD66b was higher on sputum neutrophils in control subjects, with a similar, but non-significant trend in the COPD subjects (Figure 2H). There was no difference in CD54 expression between blood and sputum neutrophils in the controls but expression was lower on sputum neutrophils compared to blood in the COPD group.

### Neutrophil markers following rhinovirus infection

**Figure 3:**
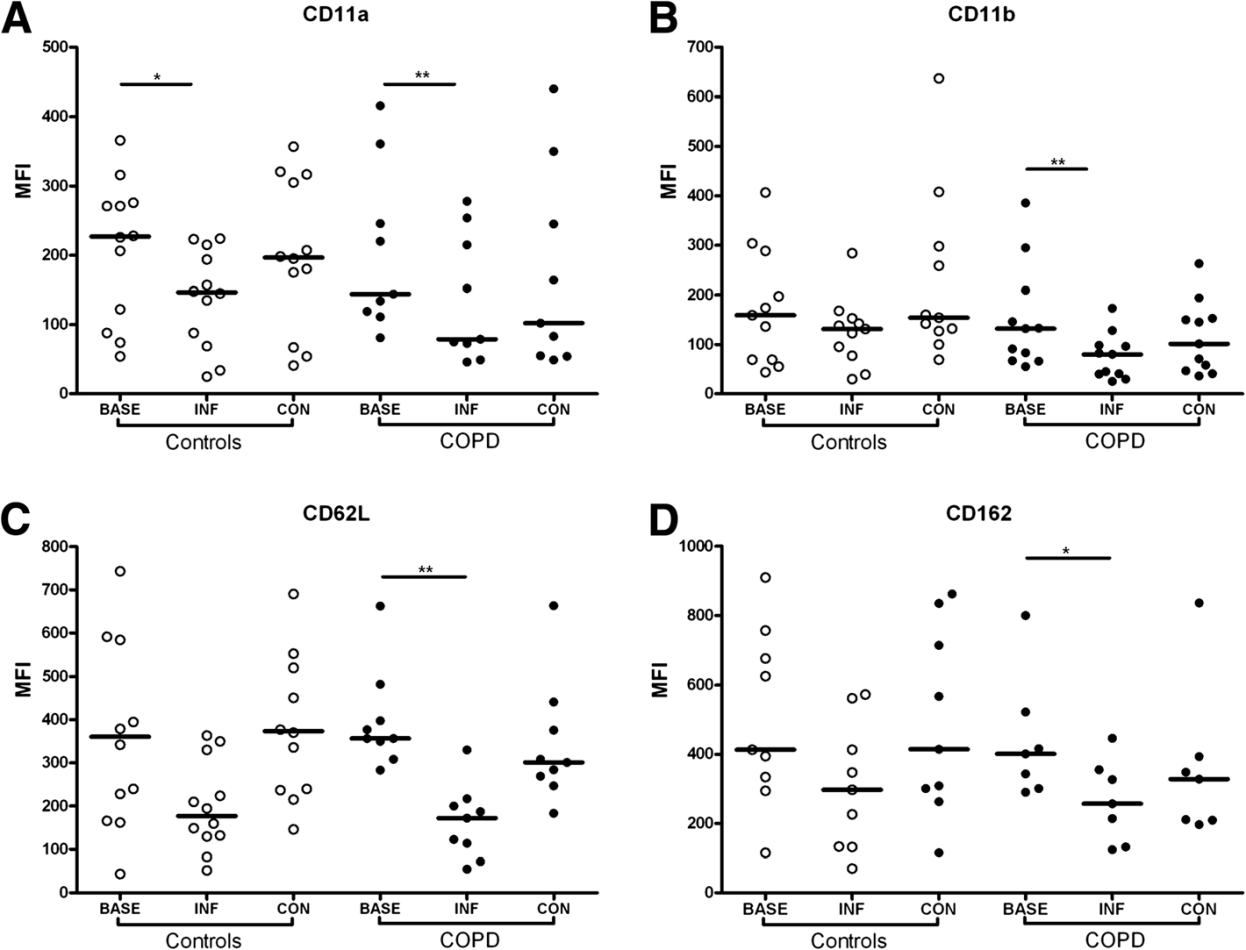
**Expression of adhesion molecules on blood neutrophils at baseline, during rhinovirus infection and at convalescence in COPD subjects and non-obstructed smokers.** CD11a **(Panel A)**, CD11b **(Panel B)**, CD162 **(Panel C)** and CD62L **(Panel D)**. * P < 0.05 vs. baseline, ** P < 0.01 vs. baseline.

Following rhinovirus infection there was no significant change in expression of surface markers on sputum neutrophils (data not shown). On blood neutrophils expression of CD11a, CD11b, CD62L and CD162 was reduced compared to baseline values on days 5–12 post-inoculation, followed by recovery to baseline levels. The nadir of marker expression from these 3 time points was chosen as the ‘infection’ time point and compared to baseline (pre-infection) and convalescence (day 42) values. There was a significant fall in CD11a expression at infection compared to baseline in both groups (P < 0.05) (Figure 3A). Expression of CD11b, CD62L and CD162 on blood neutrophils was significantly reduced at infection compared to baseline in the COPD group but not in the control subjects (Figure 3B-3D). There was a trend towards increased expression of CD54 after infection but this was not statistically significant. Expression of CD31, CD63 and CD66 on blood neutrophils did not change after infection in either group (data not shown).

### Relationships between neutrophil markers and clinical and inflammatory parameters

**Figure 4:**
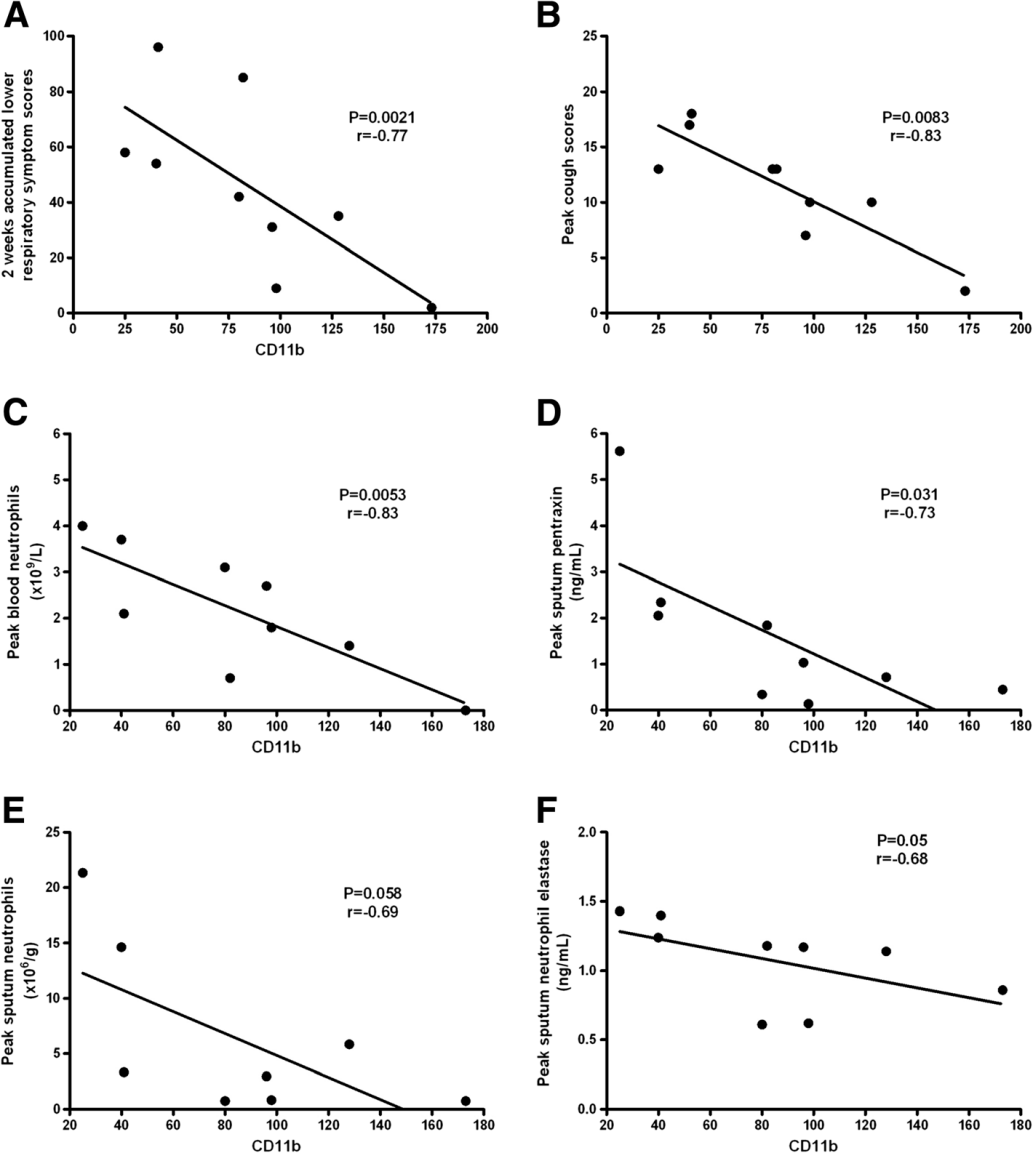
**Correlations between CD11b expression on blood neutrophils during rhinovirus infection with clinical and inflammatory parameters in COPD subjects. Panel A**. Two week aggregated lower respiratory symptoms scores. **Panel B**. Peak cough scores. **Panel C**. Peak blood neutrophil counts. **Panel D**. Peak sputum pentraxin levels. **Panel E**. Peak sputum neutrophils. **Panel F**. Peak sputum neutrophil elastase levels.

We analysed relationships between expression of neutrophil markers and clinical and inflammatory markers following rhinovirus infection. In the COPD subjects lower CD11b levels on blood neutrophils at infection were associated with more symptoms, higher blood neutrophils and higher levels of sputum neutrophil markers (Figure 4). CD11b correlated inversely with cumulative 2 week acute phase lower respiratory symptom scores (p = 0.021, r = −0.77), cough scores (p = 0.0083, r = −0.83), peak blood neutrophils (p = 0.0053, r = −0.83) and sputum pentraxin (p = 0.031, r = −0.73) levels. There were trends towards correlations with sputum neutrophil elastase (p = 0.05, r = −0.68), sputum neutrophils (p = 0.058, r = −0.69) and sputum IL-6 (p = 0.076, r = −0.63). There were no correlations in the control subjects and there were no correlations between any other neutrophil markers and clinical and inflammatory parameters at infection.

## Discussion

This is the first study to prospectively study neutrophil adhesion molecules and activation markers in COPD exacerbations. Following rhinovirus infection CD11b, CD162 and CD62L levels were significantly reduced on circulating neutrophils in COPD subjects, and CD11b levels correlated with clinical and inflammatory parameters at exacerbation. The results indicate a role for these adhesion molecules in the recruitment of neutrophils to the lungs in COPD exacerbations and may represent potential therapeutic targets.

COPD exacerbations are associated with neutrophilic inflammation [3,4], and neutrophil migration from the blood is mediated by adhesion molecules that are well characterised *in vitro* but few studies have examined their role in COPD exacerbations *in vivo*. Pharmaceutical agents targeting these molecules are under development as novel anti-inflammatory agents in asthma and COPD [6], and therefore a better understanding of their role in these diseases is required. We have previously reported that experimental rhinovirus infection induces increases in airway neutrophils in subjects with COPD [4]. Following on from these observations we examined expression of neutrophil adhesion molecules and activation markers in experimental rhinovirus infection in COPD subjects and a control group of non-obstructed smokers.

We initially examined neutrophil markers at baseline prior to infection and there were no differences in expression in neutrophil markers between the groups. Previous studies have reported increased CD162 [11], no differences in CD11a and CD62L [12-15] and both increased [12,14,16,17] and unchanged [14,15] CD11b on neutrophils in COPD. These discrepant results are likely due to variations between studies in severity of COPD, different control groups and different methods of neutrophil isolation. The COPD subjects in our study had moderate COPD (GOLD stage II) and the comparator group were similarly aged smokers without airway obstruction. Therefore the exclusion of patients with severe COPD and the choice of smokers as the comparator group may account for the lack of differences between the groups. Comparing marker expression on blood and sputum neutrophils we report up-regulation of CD11b, CD63 and CD66 and down-regulation of CD11a, CD31, CD162 and CD62L on sputum neutrophils. Although CD11b is an adhesion molecule it is released from granules in neutrophils on activation and therefore unlike the other adhesion molecules it is upregulated following neutrophil migration. Our results are similar to those reported in allergy [18], asthma [19], bronchiolitis [20] and sarcoidosis [10]. In COPD only CD11b has been examined previously and increased expression on sputum neutrophils was reported [17]. CD162 is down-regulated upon neutrophil stimulation *in vitro*[21,22] but has not been examined *in vivo* and therefore this is the first study to report down-regulation of CD162 following neutrophil migration to the lung *in vivo*. Following rhinovirus infection there were no changes in neutrophil marker expression on sputum neutrophils. These findings imply that changes in neutrophil surface markers following migration from the blood to the lung are an ‘all or nothing’ phenomenom and not specific to any particular disease process [10,18]. Therefore markers on sputum neutrophils are unlikely to be helpful as diagnostic or prognostic markers in airway diseases.

In contrast there were consistent changes in marker expression on blood neutrophils following rhinovirus infection. Only one study has examined neutrophil adhesion molecules in COPD exacerbations and this study reported reduced CD11a and CD11b but not CD62L on blood neutrophils in exacerbated patients [12]. Studies of neutrophils in virus infections have had conflicting results. Peripheral blood neutrophils collected during RSV infections in infants showed no differences in CD11a, CD11b or CD62L compared to controls [20]. In children with viral wheeze CD62L was reduced but not CD11b [23], and in adults with influenza infection CD11b was increased [24]. These studies were all cross-sectional with different subjects as controls, making interpretation of the dynamics of neutrophil responses difficult. Our study is the first to prospectively study marker expression within the same subjects prior to and during the course of virus infections. We hypothesised that neutrophils expressing higher levels of adhesion molecules would preferentially migrate to the lungs following infection, and therefore the cells remaining would consist of a population with lower surface levels of adhesion molecules. Following rhinovirus infection CD11a on peripheral blood neutrophils was reduced in both COPD and non-COPD subjects, and CD11b, CD62L and CD162 were significantly reduced in COPD subjects only. These data favour our hypothesis and suggest that these adhesion molecules are key to neutrophil recruitment to the lungs following virus infection in COPD. There was no change in CD31, CD66b and CD63 expression on blood neutrophils post-infection, consistent with their role in endothelial transmigration and as neutrophil activation markers respectively. There was a trend towards increased CD54 expression on blood neutrophils post-infection. A role of CD54 in neutrophil migration is unlikely, given our current state of knowledge, as it has no known ligands on endothelial cells, but it has been reported up-regulated on peripheral blood neutrophils in RSV infection [20], following exposure to endotoxin [25] and in sarcoidosis [10], and therefore may be up-regulated in response to systemic inflammation. Further studies are needed to determine whether CD54 is upregulated in more severe exacerbations or bacterial exacerbations and whether it plays a role in neutrophil chemotaxis

There were significant inverse relationships between CD11b expression on blood neutrophils in the COPD subjects and respiratory symptoms and inflammatory markers. Lower levels of blood CD11b post-infection were associated with more symptoms and greater neutrophilic inflammation in both blood and sputum. These data suggest that CD11b is closely linked to virus-induced neutrophilic inflammation in COPD exacerbations and CD11b has potential as both a marker and therapeutic target in COPD exacerbations. In an ozone-induced model of airway inflammation the CXCR2 antagonist SB-656933 inhibited CXCL1-induced CD11b expression on peripheral blood neutrophils and reduced neutrophilic inflammation in sputum [26]. Our data suggests that this approach may also have potential as a treatment for virus-induced COPD exacerbations. Given our recent observation that secondary bacterial infections occurred in 60% of COPD subjects following experimental rhinovirus infection, and that virus-induced neutrophilic airway inflammation was implicated in precipitating bacterial overgrowth via neutrophil elastase-mediated degradation of anti-microbial peptides, therapeutic approaches that reduce neutrophil migration and activation might have the attractive potential of preventing secondary bacterial infections [7].

Our study has a number of limitations that must be considered when interpreting the results. The number of subjects studied was relatively small and experimental rhinovirus infection is limited to patients with moderate COPD, and therefore may not be relevant to more severe disease. Experimental infection studies will always be limited by these factors but we believe that such studies are a useful tool to investigate mechanisms in COPD exacerbations and generate hypotheses that warrant further investigation in naturally-occurring exacerbations.

The surface markers we used to identify sputum neutrophils may not have excluded other cell types particularly eosinophils. However eosinophils represented <2% of sputum cells in these subjects and therefore are unlikely to have had a major impact on the results [4]. Moreover the results from the blood neutrophils are likely to be valid as purified neutrophils were used.

In conclusion this is the first study to prospectively study neutrophil marker expression in experimental rhinovirus infection. We observed reduced expression of the adhesion molecules CD11a, CD11b, CD62L and CD162 on peripheral blood neutrophils following rhinovirus infection in COPD subjects, and correlations between CD11b and exacerbation severity. Further studies investigating the role of adhesion molecules in other virus-induced disease such as asthma exacerbations, and interventions targeting neutrophil adhesion molecules are warranted.

## Abbreviations

COPD: Chronic obstructive pulmonary disease; CD62L: L-selectin; PSGL-1, CD162: P-selectin glycoprotein ligand-1; LFA-1, CD11a: Lymphocyte function-associated antigen-1; MAC-1, CD11b: Macrophage associated antigen-1; PE: Phycoerythrin; FITC: Fluorescein isothiocyanate; QR: Quantum red; MFI: Mean fluorescence intensity.

## Competing interests

The authors declare they have no competing interests.

## Authors’ contributions

PM, SLJ, SDM and LAS designed the study. PM, SDM, LAS, MC, KKG, AT, VLS, AP, SE, OM, MJ all contributed to the clinical and laboratory work for the study. PM and SLJ analysed the data and wrote the paper. All authors read and approved the final manuscript.
